# Effect of Incidence Angle on Entropy Generation in the Boundary Layers on the Blade Suction Surface in a Compressor Cascade

**DOI:** 10.3390/e21111049

**Published:** 2019-10-27

**Authors:** Lei Shi, Hongwei Ma

**Affiliations:** School of Energy and Power Engineering, Beihang University, Beijing 100191, China

**Keywords:** entropy generation, transitional boundary layer, turbulence, incidence angle, particle image velocimetry, compressor cascade

## Abstract

The entropy generation that occurs within boundary layers over a C4 blade at a Reynolds number of 24,000 and incidence angles (i) of 0°, 2.5°, 5°, 7.5°, and 10° are investigated experimentally using a particle image velocimetry (PIV) technique. To clarify the entropy generation process, the distribution of the entropy generation rates (EGR) and the unsteady flow structures within the PIV snapshots are analyzed. The results identify that for a higher incidence angle, the separation and transition occur further upstream, and the entropy generation in the boundary layer increases. When the separation takes place at the aft portion of the blade, the integral EGR decrease near the leading edge, remain minimal values in the middle portion of the blade, and increase sharply in the vicinity of the mean transition. More than 35% of the entropy generation is generated at the region downstream of the mean transition. When the separation occurs at the fore portion of the blade, the contributions of mean-flow viscous dissipation decrease to less than 20%. The entropy generation with elevated value can be detected over the entire blade. The entire integral entropy generation in the boundary layer increases sharply when the laminar separation bubble moves upstream to the leading edge.

## 1. Introduction

Compressors that operated in a low-speed/high-altitude condition, experience a flow regime strongly influenced by the separated shear layer. When the separated shear layer transitions to turbulence and reattaches, a laminar separation bubble (LSB) is generated [1,2,3,4,5]. During the process of flow separation, transition, and reattachment, entropy is inevitably generated by fluid friction and turbulent dissipation. A key to improving efficiency of compressors is the minimization of the loss [6], namely to minimize the entropy generation [7,8,9,10]. Therefore, the process of the entropy generation in the boundary layer should be figured out. For this purpose, the approximation method of the entropy generation rates (EGR) [11,12,13,14] is a useful tool, and provides detailed information about where and how entropy is locally generated.

Over the last five decades, there has been increasing interest in studying the EGR in wall-bounded flows. For pure laminar flows and developed turbulent flows, Bejan [7], Adeyinka [15], McEligot [16] studied the viscous contribution of the mean flow to the EGR, and stated that most of the EGR take place in the very thin viscous sublayer. However, the transition process would lead to significant increases of turbulent dissipation and the entropy generation. The EGR would be seriously underestimated when the effect of turbulence dissipation is totally ignored. To solve this problem, Rotta [17] added turbulent dissipation, and gave the formula of complete pointwise EGR considering both the viscous and turbulent effect. Walsh and McEligot [18,19] utilized direct numerical simulation (DNS) results to quantify the EGR during the transition from laminar to turbulent flow for several pressure gradients, including zero pressure gradient, favorable pressure gradient, and adverse pressure gradient. Based on Walsh and McEligot’s studies, Skifton [20] performed particle image velocimetry (PIV) measurements with similar flow configurations on the plate. The PIV results and DNS results showed very similar trends that the turbulent contributions of the EGR traded off with the viscous contributions through the transition process and beyond. Downstream of the transition, the turbulent contributions become the dominant term in the total EGR. Consequently, the location and extent of the mean transition play vital roles in the process of the entropy generation in the boundary layer.

It has been established in previous investigations [21,22,23] that the mean transition is closely related to the formation and evolution of vortical structures within the LSB. Experimental studies [23,24,25,26] over airfoils and turbomachinery blades at low Reynolds numbers clarified that the boundary layer often remains laminar in the fore part, and becomes susceptible to separation past the suction peak under an adverse pressure gradient. In the vicinity of the separation, small-amplitude disturbances are amplified. The amplification of disturbance leads to the shear layer vortices roll-up and shedding. It is found that [27,28] vortex dynamics is highly dependent on Angle of attack, the Reynolds number and the degree of freestream turbulence. Burgman and Schroder [24] performed time-resolved PIV measurements over an SD7003 airfoil at different flow conditions, and clarified that the separation, transition, and reattachment were governed by the angle of attack. Recently, Lambert and Yarusevych [29] experimentally investigated the dynamics of coherent structures that develop within the LSB over a NACA 0018 airfoil at a Reynolds number of 100,000 and angles of attack of 0°, 5°, 8°, and 10° using a high-speed flow visualization technique and embedded microphones. Their results showed that vortex rolled up and salient aspects of vortex development took place within the same regions when scaled by the LSB length, and the normalized transition location by the LSB length is essentially invariant to angle of attack. Inspired by these specific works, a question was arose that whether the entropy generation process on the blade suction surface is invariant to incidence angle when scaled by the LSB length.

In this study, the entropy generation that occurs in the boundary layer over a C4 blade at a Reynolds number of 24,000 and incidence angles of 0°, 2.5°, 5°, 7.5°, and 10° are investigated experimentally using particle image velocimetry (PIV) measurements in a compressor cascade. Based on the high-resolution flow field of PIV results, the characterization of the boundary layer, vortex development within the boundary layer are represented. The EGR distribution within the field of view (FOV) and the corresponding unsteady flow structures are analyzed to clarify the entropy generation process for different incidence angles. Hopefully, this work could be helpful to understand the loss mechanism in the boundary layer and to provide guidance for separation control and performance improvement.

This paper is organized as follows. First, the experimental methods are described. Subsequently, the approximation method for the EGR in the boundary layer is introduced. In the following part, the time-averaged and instantaneous flow field are shown and the entropy generation process is analyzed. Finally, the findings are summarized and conclusions are drawn. 

## 2. Experimental Method

**Figure 1:**
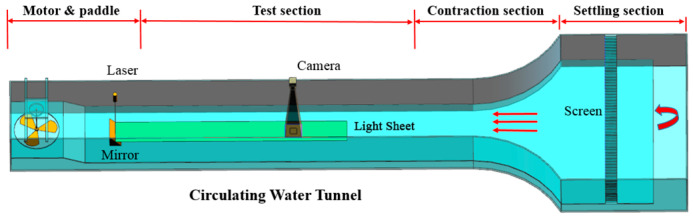
Schematic of the water tunnel and particle image velocimetry (PIV) configuration.

Measurements were performed in a transparent circulating water tunnel of Beihang University, as shown in Figure 1. The water tunnel is 6.8 m long with a 700 mm (width) × 500 mm (depth) test section. In the test section, the freestream velocity was set to 0.1 m/s during the measurement. For the conditions investigated, the turbulence intensity in the test section was less than 1%, and the Reynolds number based on the chord length and inlet velocity was 24,000.

**Figure 2:**
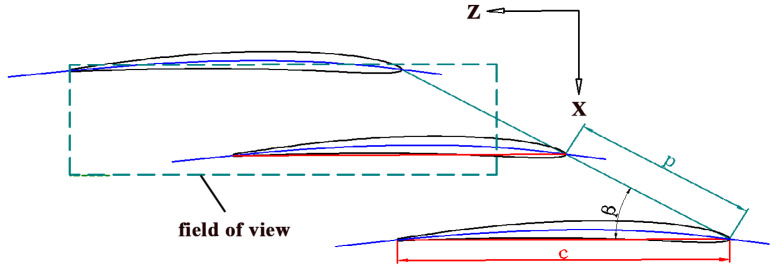
Schematic of the compressor cascade and PIV field of view.

The compressor cascade, as schematically shown in Figure 2, was placed in the test section. The z and x coordinates in Figure 2 correspond to the streamwise and wall-normal directions, respectively. The compressor cascade consists of 12 C4 base-profile blades, tip wall, hub wall, and profile sidewalls. The tip clearance was set to zero. The parameters of the cascade and the test conditions are listed in Table 1.

An advanced commercial PIV system was employed to characterize the flow development in the boundary layer. The PIV measurement was performed in the configurations shown in Figure 1. The flow was illuminated by a laser-sheet generated by a dual-cavity ND: YAG pulse laser device (200 mJ /pulse at a 15 Hz repetition rate, 532 nm wavelength). The thickness of the laser-sheet was about 0.5 mm. The digital imaging was conducted by a 12 bit frame-straddling based CCD camera (2072 × 2072 pixels) with a macro lens. The FOV covered approximately one and a half chord length in the streamwise direction and one pitch length in the wall-normal direction.

In order to eliminate the effect of movement along the spanwise direction on the measurement results, measurements were performed on the mid-span plane of the blade. During the measurements, a mirror was placed downstream of the cascade. The laser lighted up the measurement plate through the mirror reflection.

1000 pairs of snapshots were acquired at a sampling rate of 10 Hz. A median-subtraction filter algorithm was applied to all images to remove non-uniformities in the background light intensity. The convergence process of the velocity profiles on the blade suction surface at z/C = 1.00 shows that 1000 image pairs are enough to ensure the convergence of the results, which is not shown for conciseness. The cross-correlation of each image pair was calculated by the software MicroVec V3 with the two-pass PIV interrogation algorithm. The interrogation region was 64 × 16 pixels in the first pass while 32 × 8 pixels in the second pass. Consequently, the spatial distance between the adjacent vectors is 1.15 mm × 0.29 mm. The relative deviation from the average vector was calculated in order to eliminate questionable vectors, and the relative deviation threshold was set to be 50%. According to the analyses of Raffle [30] and Westerweel [31], the total random error is about 0.1 pixel when the particle image displacement is larger than 0.6 pixel. Taking other uncertainties into consideration, the total random error is estimated as 0.15 pixels. The experimental uncertainty for the instantaneous velocity is estimated to be 3.5% of the maximum velocity. 

## 3. Approximation Method for EGR

In a two-dimensional flow with turbulent fluctuations, the EGR per unit volume $S^{\prime \prime \prime }$ can be expressed as the sum of two terms [17,19,20,32]: the contributions from the mean-flow viscous dissipation μΦ and the turbulent dissipation ρε,

(1) $$S^{\prime \prime \prime }=\frac{\mu \Phi +\rho \varepsilon }{T}$$

The first term μΦ in Equation (1) represents the fraction of the dissipation which is due to the mean velocity gradient and is called the dissipation of mean flow kinetic energy, and may be expressed as [7]

(2) $$\mu \Phi =2\mu [{\left(\frac{\partial \bar{w}}{\partial z}\right)}^{2}+{\left(\frac{\partial \bar{u}}{\partial x}\right)}^{2}]+\mu {\left(\frac{\partial \bar{w}}{\partial x}+\frac{\partial \bar{u}}{\partial z}\right)}^{2}$$

The second term ρε in Equation (1) highlights the dissipation of turbulence kinetic energy into thermal energy, and could be expressed as [33]

(3) $$\rho \varepsilon =2\mu [{\left(\frac{\partial w^{\prime }}{\partial z}\right)}^{2}+{\left(\frac{\partial u^{\prime }}{\partial x}\right)}^{2}]+\mu {\left(\frac{\partial w^{\prime }}{\partial x}+\frac{\partial u^{\prime }}{\partial z}\right)}^{2}$$

After the introduction of boundary layer simplification [17], Walsh [19] integral the EGR per unit volume $S^{\prime \prime \prime }$ in wall-normal direction, and yielded the EGR per unit area,

(4) $$\begin{array}{l} S\prime \prime \{\delta \}=\int_{0}^{\delta }S^{\prime \prime \prime }\mathrm{dx}\approx \frac{\rho }{T}[\nu {\int_{0}^{\delta }\left(\frac{\partial \bar{w}}{\partial x}\right)}^{2}dx-\int_{0}^{\delta }\left(\bar{u^{\prime }\;w\prime }\right)(\frac{\partial \bar{w}}{\partial x})dx-\int_{0}^{\delta }\left[(\bar{{w^{\prime }}^{2}}-\bar{{u^{\prime }}^{2}})\right](\frac{\partial \bar{w}}{\partial z})dx\\ \;(1)\;(2)\;(3)\;\\ -\frac{d}{dz}\int_{0}^{\delta }\frac{1}{2}\bar{w}(\bar{q^{2}})dx-\frac{1}{2}\bar{u_{\delta }[(w_{\delta }{}^{2})+(u_{\delta }{}^{2})]}-w_{\delta }p_{\delta }]\\ \;(4)\;(5)\;(6)\; \end{array}$$

Terms 1–6 in Equation (4) represent the contributions of the mean viscous effect, the Reynolds shear stress production, the Reynolds normal stress production, the turbulent energy flux, the turbulent diffusion and the pressure diffusion, respectively. As found by Walsh [19], the last two terms (terms 5 and 6) are minimal comparing to the total EGR, and can be neglected. In this paper, we will discuss the first four terms based on the PIV instantaneous snapshots. 

## 4. Results and Discussions

### 4.1. Separation Bubble Characterization

**Figure 3:**
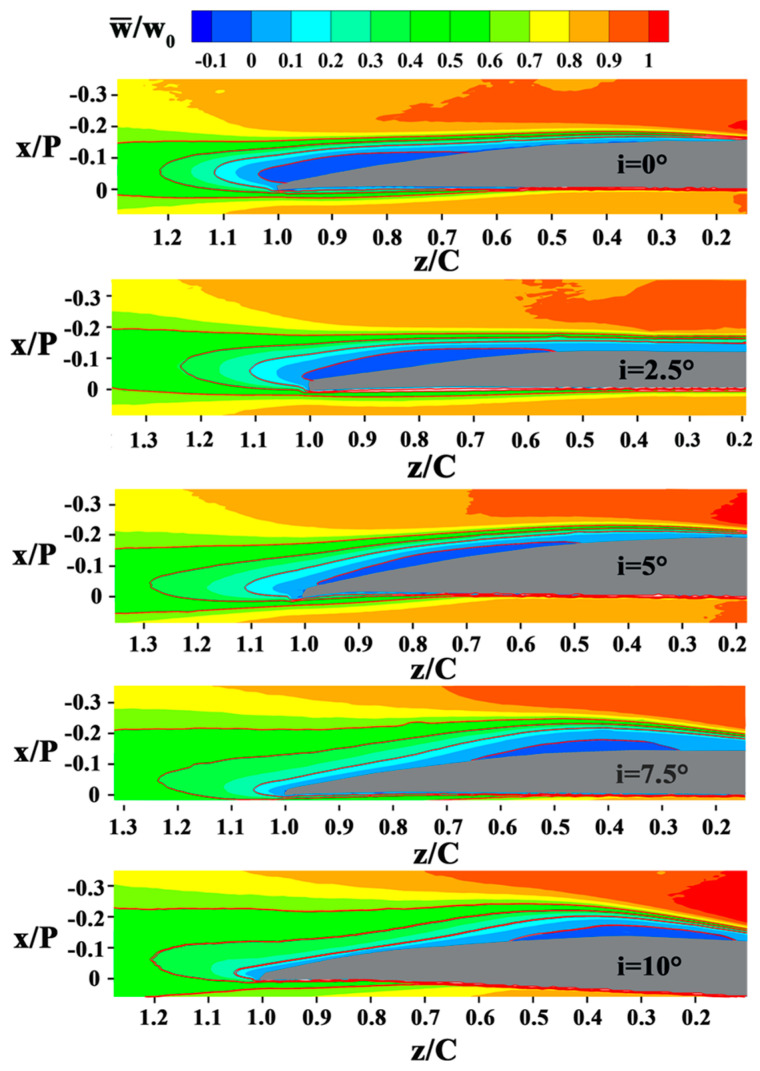
Time-averaged streamwise velocity normalized by the inlet mainstream velocity w_0_ for five incidence angles. The red contour lines in each figure show the normalized streamwise velocity values of 0, 0.2, 0.4, and 0.6.

The distribution of time-averaged streamwise velocity is demonstrated in Figure 3. The time-averaged streamwise velocity is normalized by the mainstream velocity. The separation point (z_s_) is identified as the position where a null time-averaged wall-normal velocity gradient has been detected. Separation is found to be steady using this method. Mean transition onset [24] is defined as the point (z_t_) where the growth rate of the Reynolds shear stress deviates from the exponential growth. The reattachment point (z_r_) is identified as the same method with that of the separation point (z_s_). Since the reattachment occurs downstream of the separation, the two positions with zero values of (∂w¯∂Χ)Χ=0 can be distinguished.

**Figure 4:**
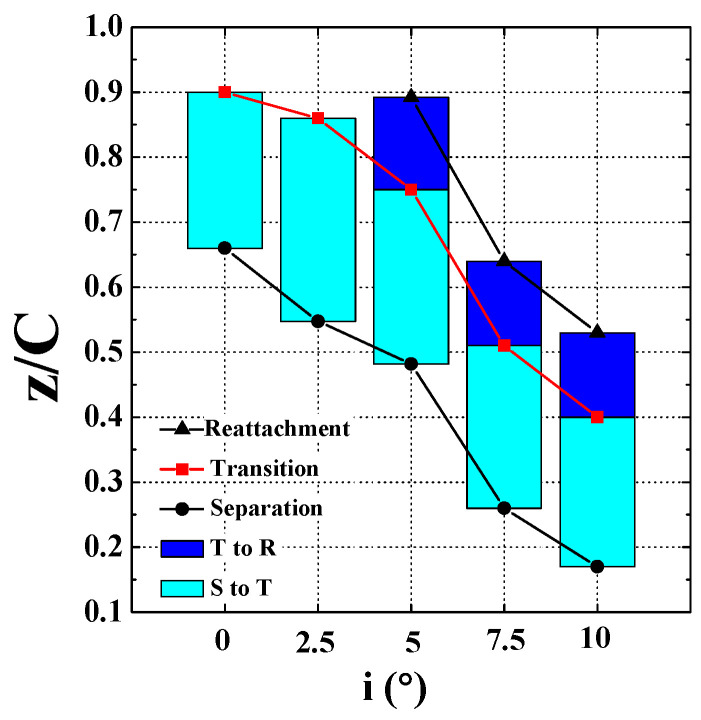
Variations in mean separation, transition and reattachment locations with incidence angle.

The separation advances with increasing incidence angle. The separation point moves forward from z/C = 0.66 at i = 0° to z/C = 0.48 at i = 5°, and z/C = 0.17 at i = 10°. The LSB also reattached early from z/C = 0.89 at i = 5° to z/C = 0.64 at i = 7.5°, and z/C = 0.53 at i = 10°. At low incidence angles (i = 0° and i = 2.5°), the separated shear layers do not reattach. The development of the locations of the mean separation, transition, and reattachment with the incidence angle is summarized in Figure 4. The LSB moves forward and becomes shorter as the incidence angle increases from i = 0° to i = 10°, similar to the variation tendency reported in the previous investigations [21,24,29,34,35]. The distance between the separation and the transition normalized by the bubble length at i = 5° is 0.65, similar to 0.66 at i = 7.5° and 0.64 at i = 10°. The normalized transition location takes place at approximately 70% of the LSB length, which is similar to the result reported in the airfoil flow study of Lambert et al [29].

### 4.2. Instantaneous Flow Fields

**Figure 5:**
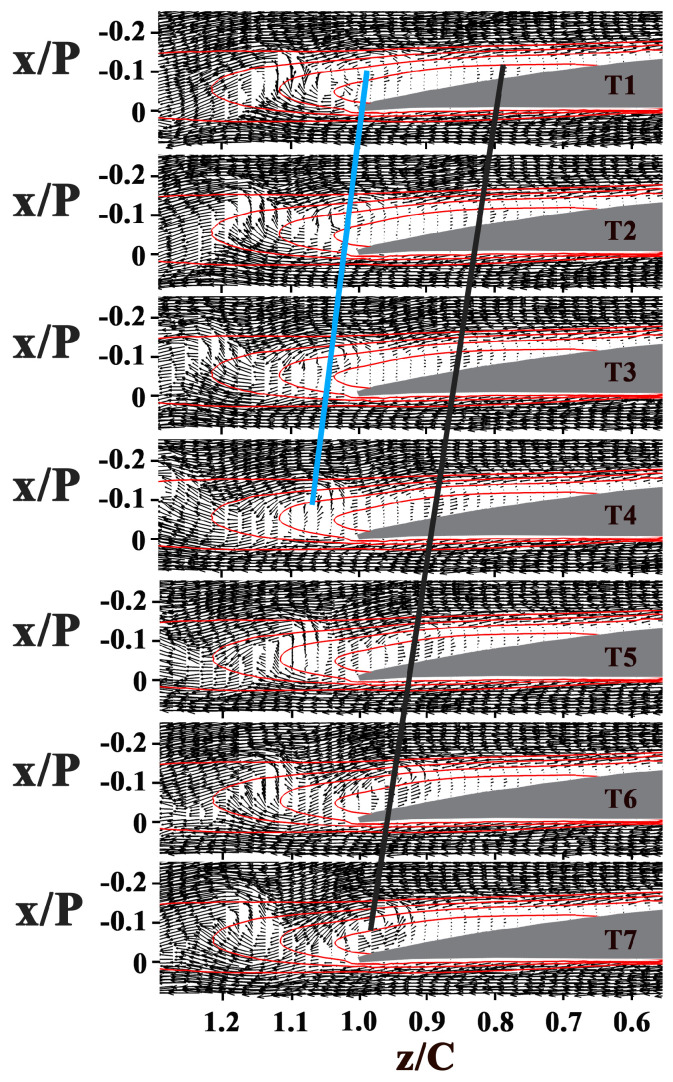
Sequence of velocity vector diagrams at i = 0°. Time interval is 0.1 s. Black and blue Lines give an indication of the positions and drift velocity of the vortices. The red contour lines in each figure show the normalized streamwise velocity values of 0, 0.2, 0.4, and 0.6.

**Figure 6:**
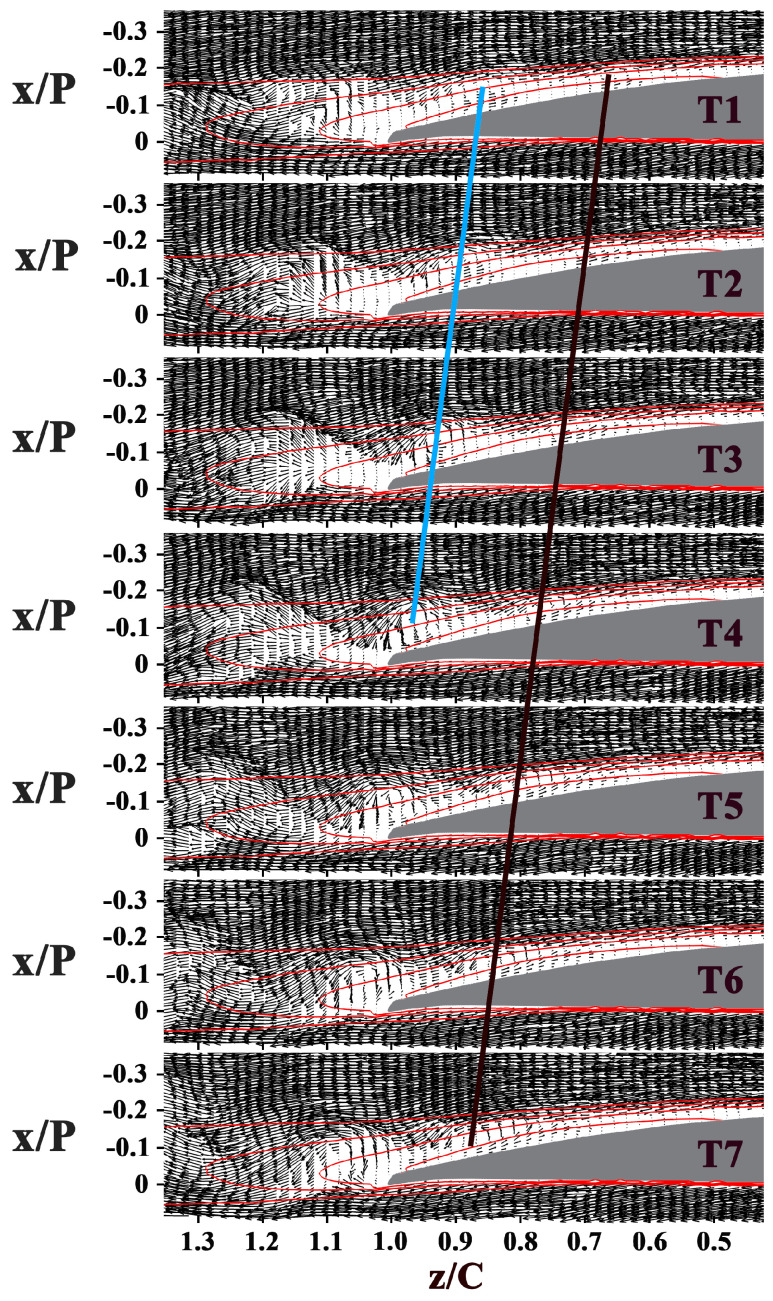
Sequence of velocity vector diagrams at i = 5°. Time interval is 0.1 s. Black and blue lines give an indication of the positions and drift velocity of the vortices. The red contour lines in each figure show the normalized streamwise velocity values of 0, 0.2, 0.4, and 0.6.

**Figure 7:**
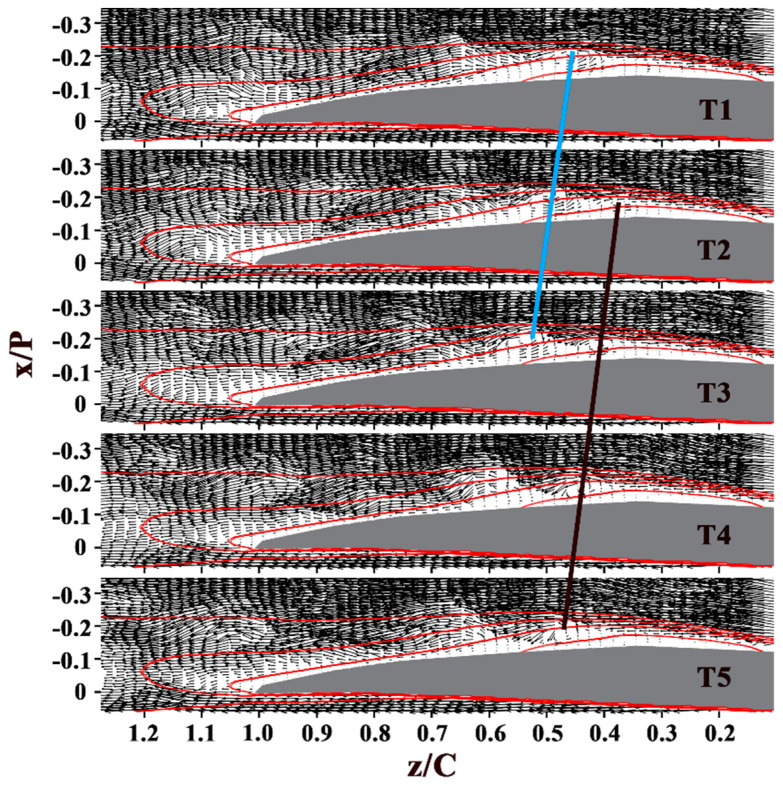
Sequence of velocity vector diagrams at i = 10°. Time interval is 0.1 s. Black and blue lines give an indication of the positions and drift velocity of the vortices. The red contour lines in each figure show the normalized streamwise velocity values of 0, 0.2, 0.4, and 0.6.

The instantaneous snapshots sequences of both the blade channel flow and the wake flow at i = 0°, 5°, and 10° are presented in Figure 5, Figure 6 and Figure 7. In these velocity vector diagrams, only every second vector is plotted for clarity. 

As the disturbances grow, vortices can be found upstream of the mean transition. The time-space distribution of instantaneous vector maps in Figure 5 captures the development of two consecutive shear layer vortices, labeled V1 and V2 at i = 0°. Inclined lines superimposed on the plots connect the core of these vortices, black and blue lines follow the V1 and V2 vortical perturbation, respectively. The slope of the lines are related to vortices convective velocity. The results illustrate that V1 originating at 0.74 < z/C < 0.80 at T1 moves up to 0.92 < z/C < 1.00 at T7. Along this motion, the vortex lifts up: it originates close to the blade suction surface and moves upwards at the trailing edge. The position of V2 at T1 can be speculated as the position of V1 in the next snapshot of T7. Thus, V1 continues its movement as V2 at T1 (0.95 < z/C < 1.05) for periodicity. As indicated by the slope flattened of the downstream line, V1 decelerates. V1 gets deformed past the trailing edge. Eventually, V1 loses its coherence and cannot be detected at 10% of the chord length downstream of the blade end.

When the incidence angle grows, the separation bubble moves forward (as shown in Figure 6 and Figure 7), so does the location where the vortices originate, advancing from z/C = 0.78 at i = 0° to z/C = 0.63 at i = 5°, and z/C = 0.37 at i = 10°. The wavelength of the vortices decreases. The vortex slightly decelerates past the trailing edge at i = 5° while the drift velocity of vortices remain the same past the mean transition at i = 10°.

Based on these results, several key observations can be made. First, at large incidence angle (i = 7.5° and i = 10°), the vortices with lower strength and smaller scale get deformed past the mean transition, and break down into small structures past the mean reattachment. Second, the shear layer roll-up vortices retain sufficient strength and coherence at the trailing edge and even past the trailing edge at i = 0°. Third, the vortices interact with the wake flow past the trailing edge at low incidence angles (i = 0°, i = 2.5°, and i = 5°). During the interaction, the vortices decelerate. 

### 4.3. Entropy Analysis

**Figure 8:**
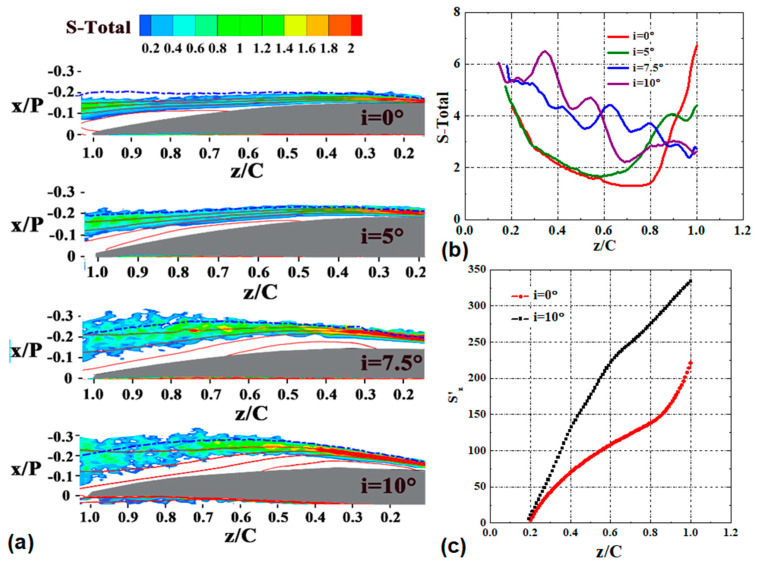
The variation of the entropy generation rates (EGR) with incidence angle. (**a**) Distribution of the EGR S’’’ for four incidence angles. The red contour lines in each figure show the normalized streamwise velocity values of 0, 0.2, 0.4, and 0.6. The blue dash line represents the boundary layer where streamwise velocity is 90% of the local mainstream streamwise velocity. (**b**) The development of the EGR S’’{δ} along the streamwise direction. (**c**) The development of the integral entropy generation S’_z_ along the streamwise direction.

**Figure 9:**
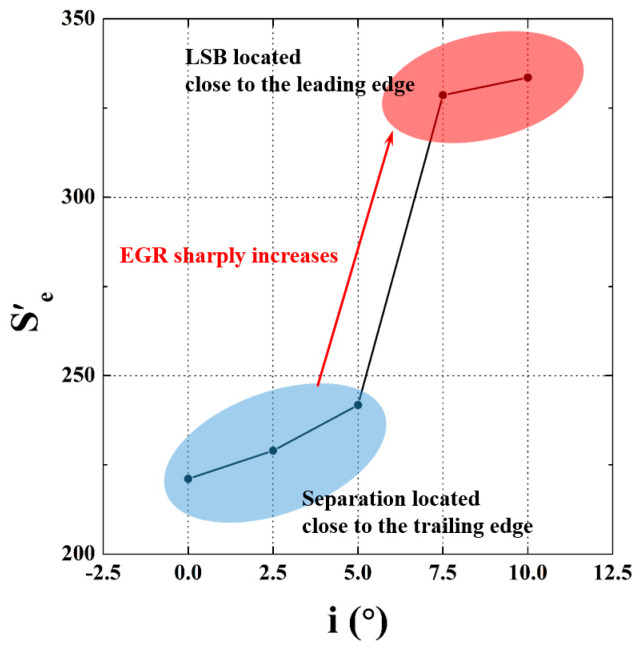
The entire EGR S’_e_ in the boundary layer with incidence angle.

The distribution of the EGR and the integration of the EGR in the boundary layer are illustrated in Figure 8. Terms 1–4 in Equation (4) are normalized by the cube of the inlet mainstream velocity w_0_. Since the distribution of the EGR at i = 2.5° is similar to that of i = 0°, the result of this case is not shown for conciseness. The distribution of the EGR shows that the entropy generation get elevated within the shear layer in the fore part of the blade, and reaches the maximum in the vicinity of the reattachment. Conversely, the EGR are practically null in the dead water region within the separation bubble. Figure 9 shows the entire EGR S’_e_ in the boundary layer with incidence angle. The entire EGR S’_e_ were calculated by integrating the EGR per unit area S’’{δ} in the entire streamwise range investigated. The entire EGR S’_e_ sharply increases when the LSB moves to the leading edge at i = 7.5°. The entire EGR S’_e_ at i = 10° are one and a half times as large as their counterparts at i = 0°. Therefore, based on the relative magnitude of the entire EGR S’_e_ and the position of the LSB as well, the cases investigated here can be divided into two main parts: the low incidence angle cases (i = 0° and i = 5°) and the high incidence angle cases (i = 7.5° and i = 10°).

The obvious difference of the EGR per unit area S’’{δ} between the low incidence angle cases (i = 0° and i = 5°) and the high incidence angle cases (i = 7.5° and i = 10°) is the variation tendency along the streamwise direction. At low incidence angle cases, the EGR S’’{δ} decrease in the vicinity of z/C = 0.2, remain relatively low value in the middle portion of the blade (0.4 < z/C < 0.8), and increase sharply in the vicinity of the mean transition, as shown in Figure 8b. At high incidence angle cases, the EGR S’’{δ} fluctuate around the maximum value over the entire blade. The integral EGR S’_z_ at i = 0° and i = 10° were calculated by integrating the EGR per unit area S’’{δ} in the streamwise coordinates. More than 35% of the entropy is generated at the region downstream of the mean transition (0.8 < z/C < 1.0) at i = 0°, as shown in Figure 8c. However, at large incidence angle (i = 10°), the entropy generation accumulates with a uniform velocity.

**Figure 10:**
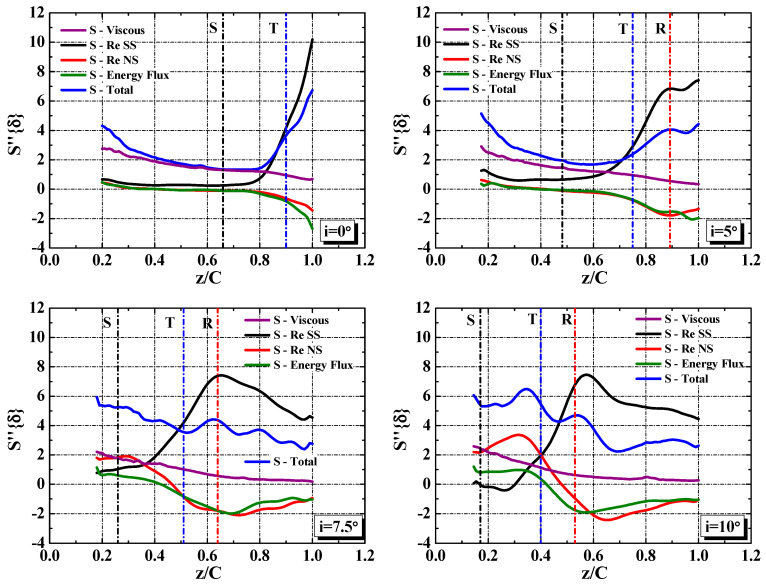
The variation of the EGR per unit area S’’{δ} along the streamwise direction with incidence angle. S, T and R in the figures represent the streamwise position of the mean separation, transition and reattachment, respectively.

To extract the dominant contribution to the entropy generation and understand the entropy generation process, the EGR per unit area S’’{δ} and its four terms are shown in Figure 10. The Reynolds shear stress production term accounts for the most at the region downstream of the mean transition. At this region, the main difference of the EGR S’’{δ} between the low incidence angle cases and the high incidence angle cases is closely related to the variation of the Reynolds shear stress production term. However, the dominant term changes for different incidence angles at the region upstream of the mean transition. At low incidence angle, the mean-flow viscous term dominates the EGR. When the incidence angle increases to i = 7.5° and i = 10°, the contributions of the Reynolds normal stress production term increase to be dominant as well. 

**Figure 11:**
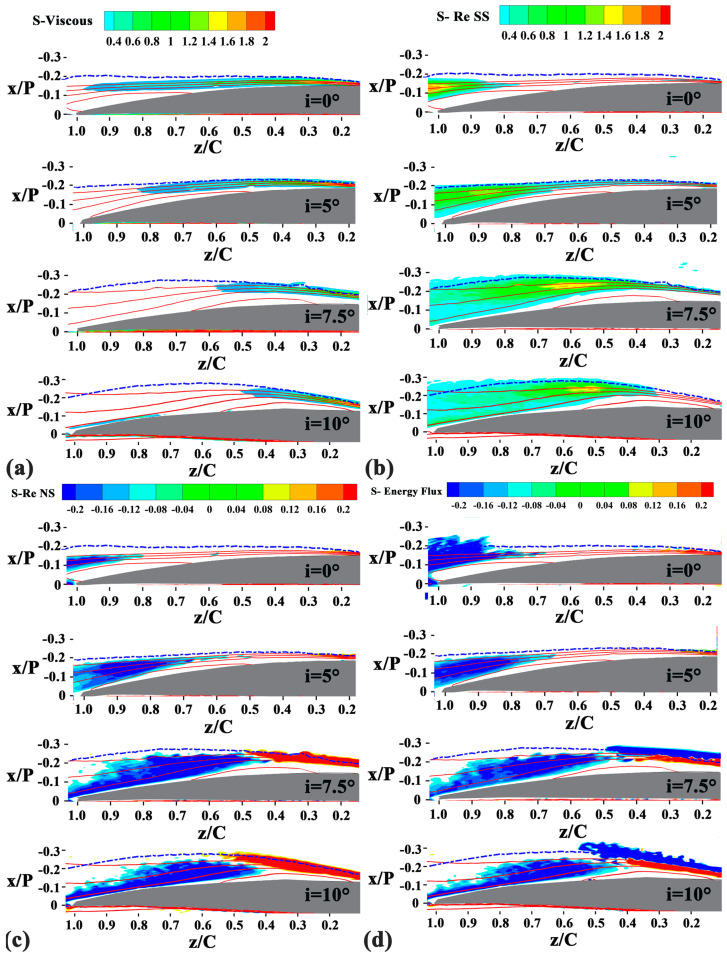
Distribution of the four terms of the EGR S’’’ at four incidence angles. (**a**) The mean viscous term, S-Viscous; (**b**) The Reynolds shear stress production term, S-Re SS; (**c**) The Reynolds normal stress production term, S-Re NS; (**d**) The energy flux term, S-Re Energy Flux. The red contour lines in each figure show the normalized streamwise velocity values of 0, 0.2, 0.4, and 0.6. The blue dash line represents the boundary layer where the streamwise velocity is 90% of the local mainstream streamwise velocity.

**Figure 12:**
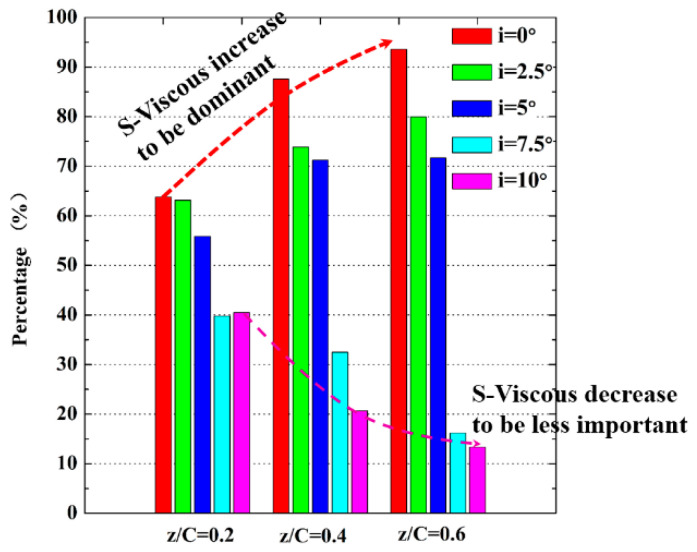
The contributions of mean-flow viscous dissipation in the overall EGR.

Figure 11 shows the distribution of the four terms of the EGR S’’’ with incidence angle. The entropy related to the mean-flow viscous dissipation generates in the fore portion of the separated shear layer, and vanishes downstream of the mean transition. Thus, the enhancement of momentum exchange due to the vortex propagation (mentioned in Section 4.2) reduces the work done by the mean flow. As the incidence angle increases, the entropy related to the mean-flow viscous dissipation decreases earlier. Figure 12 demonstrates the contribution of the mean flow viscous dissipation to the overall entropy generation. At low incidence angles, the mean viscous dissipation contributes the most part of the entropy generation. At high incidence angles, the contributions of mean viscous dissipation decrease to less than 15% at z/C = 0.6. The results show that the contributions of the mean viscous dissipation decrease to be less important when increasing the incidence angle. The results shown in Figure 12 also confirms that the ignorance of the contributions of turbulence dissipation at high incidence angle would dramatically underestimate the EGR in the vicinity of the transition, which is consistent with the findings in [19,20].

**Figure 13:**
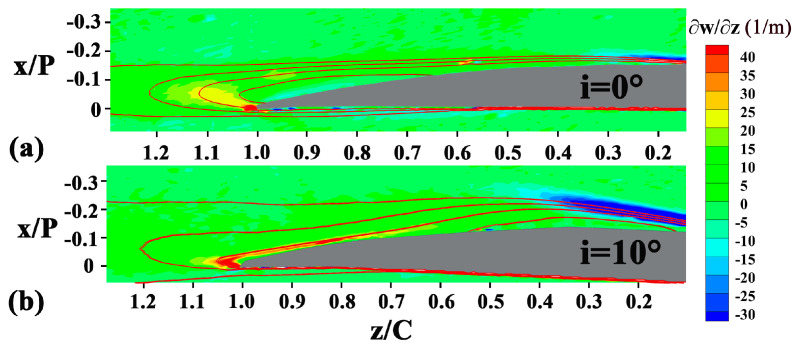
The streamwise gradient of normalized w, ∂w/∂z at: (**a**) i = 0°; (**b**) i = 10°.

In the vicinity of z/C = 0.2 at the low incidence angle cases, both the wall-normal gradient ∂w/∂x and the streamwise gradient ∂w/∂z of the streamwise velocity decrease, which leads to the decrease of the mean-flow viscous dissipation and the turbulent dissipation, and alleviates the overall entropy generation. Increasing the incidence angle to i = 7.5° and i = 10°, the Reynolds normal stress production term starts to be dominant. The advanced transition strengthens the flow deceleration, and significantly increases the negative streamwise gradient of w (∂w/∂z) as shown in Figure 13. In addition, the advanced separation also increases the Reynolds normal stress along the streamwise direction $\bar{w\prime w\prime }$. The increased ∂w/∂z and $\bar{w\prime w\prime }$ make the contribution of the Reynolds normal stress production to be dominant. 

In the aft portion of the separated shear layer (downstream of the mean transition), the magnitude of the Reynolds normal stress and the Reynolds shear stress increase to be the maximum in the vicinity of the reattachment. Consequently, the contributions of the positive Reynolds shear stress production, the negative Reynolds normal stress production term and the energy flux term increase significantly. The sum of the negative Reynolds normal stress production term and the energy flux term cannot trade off the positive Reynolds shear stress production term. Hence, the overall entropy generation significantly increases in this region. At low incidence angle cases (i = 0° and i = 2.5°), since the vortices with stronger intensity and larger scale strengthen the turbulent dissipation, the magnitude of the entropy increases with a steeper slope in the vicinity of the reattachment.

**Figure 14:**
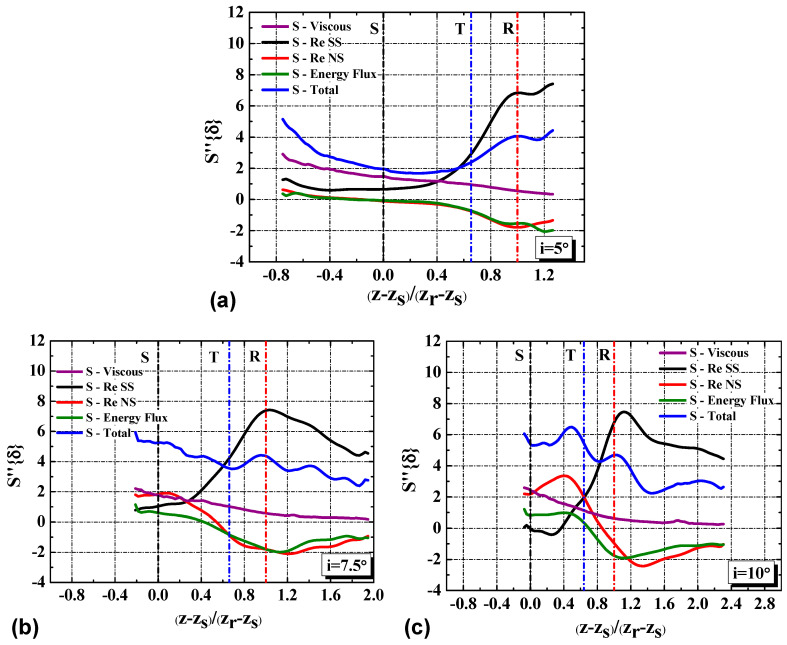
The EGR per unit area S‘’{δ} with the streamwise coordinates normalized by separation bubble length. (**a**) i=5°; (**b**) i=7.5°; (**c**) i=10°. S, T and R in the figures represent the normalized streamwise position of the mean separation, transition and reattachment, respectively.

At the region downstream of the reattachment point, the entropy generation shows different process. As mentioned in the Section 4.1, the normalized transition is essentially invariant to the incidence angle, it is thus instructive to the statistical variation of the entropy generation along the length of a separation bubble. The streamwise variation of the EGR S’’{δ} along the normalized separation bubble length is presented in Figure 14. The Reynolds shear stress production term, the Reynolds normal stress production term and the energy flux term exhibit their highest values in the vicinity of the mean reattachment point. Downstream of the mean reattachment, the Reynolds stress and velocity gradient along streamwise and wall-normal directions decrease, which alleviate the entropy generation. The locations of the largest magnitude of the overall entropy generation occur at the mean reattachment point for i = 7.5°. The counterpart location moves to downstream of the mean reattachment point at i = 5° while it moves to upstream of the mean reattachment point at i = 10°. It is being speculated that the elevated EGR at i = 5° at the blade end can be caused by the interaction of the wake and the separation vortices. As suggested by the study of Lambert et al [29], the associated aspects of vortex dynamics take place at comparable locations relative to the bubble length. The dissimilarity of the entropy generation trends across different incidence angles with respect to the position normalized by the bubble length indicates that the associated entropy generation mechanism is not simply in relation to the vortex dynamics within the separated shear layer. 

## 5. Conclusions

The entropy generation that occurs in the boundary layers over a C4 blade at Re = 24,000 and incidence angles of 0°, 2.5°, 5°, 7.5°, and 10° were investigated experimentally. PIV measurements in the compressor cascade were used to identify dominant unsteady flow structures and to understand the entropy generation process. 

At low incidence angles, the separations take place close to the blade trailing edge, and the separated boundary layer do not reattach. The shear layer roll-up vortices retain sufficient strength and coherence in the rear part of the blade and even past the trailing edge. The separation advances with increasing incidence angle. Laminar separation bubbles (LSB) are generated at i = 5°, i = 7.5°, and i = 10°. The normalized transition locations take place at approximately 70% of the LSB length. The LSB located at the fore portion of the blade at i = 7.5° and i = 10° while the LSB generated close to the blade trailing edge at i = 5°. The vortices with lower strength and smaller scales get deformed past the mean transition, and break down into small structures past the mean reattachment. 

The entire EGR S’_e_ in the boundary layer increases with increasing incidence angle. The Reynolds shear stress production term accounts for the most at the region downstream of the mean transition. When the separation takes place close to the trailing edge, the overall EGR decrease near the leading edge, remain relatively low values in the middle portion of the blade, and increase sharply in the vicinity of the mean transition. More than 35% of the entropy generation takes place in the last 20% chord of the blade. When the LSB moves to the leading edge, the entire EGR S’_e_ in the boundary layer increases sharply. The contribution of mean-flow viscous dissipation in the overall EGR decreases to less than 20% past the mean transition. The entropy generation with elevated value can be detected over the entire blade.

## Figures and Tables

**Table 1:** Cascade parameters and test condition.

Items	Details
Number of blades, N	12
Chord length, C	126.8 mm
Span, H	120 mm
Pitch, P	72 mm
Incidence angle	0°, 2.5°, 5°, 7.5°, and 10°
Stagger angle, β	30°
Tip clearance/C	0%

## Nomenclature

i	Incidence angle
x, z	Physical space coordinates
C	Chord length
H	Span
p	Pitch length
Re	Reynolds number of the inlet flow
β	Stagger angle
S’’’	Pointwise volumetric entropy generation rate
μ	Dynamic viscosity
Φ	Viscous dissipation
ρ	Fluid density
ε	Turbulent dissipation
T	Absolute temperature
$\bar{w}$	Time-averaged streamwise velocity
$\bar{u}$	Time-averaged normal velocity
w’	Streamwise velocity fluctuation
u’	Normal velocity fluctuation
δ	Boundary layer thickness
ν	Kinematic viscosity
q^2^	Sum of velocity fluctuation
()_δ_	Value at the boundary layer edge
S’’	Entropy generation rate per unit surface area
S-Total	Pointwise entropy generation rate per unit surface area
S_z_’	The total EGR calculated by integrating S’’’ in the streamwise coordinates
S_e_’	The total EGR calculated by integrating S’’’ in the entire streamwise range
S-Viscous	Entropy generation caused by the viscous effect
S-Re SS	Entropy generation caused by the Reynolds shear stress production
S-Re NS	Entropy generation caused by the Reynolds normal stress production
S-Energy Flux	Entropy generation caused by the turbulent energy flux
z_s_	The streamwise coordinate of the mean separation point
z_t_	The streamwise coordinate of the mean transition point
z_r_	The streamwise coordinate of the mean reattachment point
